# AI-Assisted Diagnosis and Decision-Making Method in Developing Countries for Osteosarcoma

**DOI:** 10.3390/healthcare10112313

**Published:** 2022-11-18

**Authors:** Haojun Tang, Hui Huang, Jun Liu, Jun Zhu, Fangfang Gou, Jia Wu

**Affiliations:** 1 School of Computer Science and Engineering, Central South University, Changsha 410083, China; 2 The First People’s Hospital of Huaihua, Huaihua 418000, China; 3 The Second People’s Hospital of Huaihua, Huaihua 418000, China; 4 Collaborative Innovation Center for Medical Artificial Intelligence and Big Data Decision Making Assistance, Hunan University of Medicine, Huaihua 418000, China; 5 Research Center for Artificial Intelligence, Monash University, Melbourne, Clayton, VIC 3800, Australia

**Keywords:** MRI image segmentation, medical system, attention, AI-assisted diagnosis, osteosarcoma

## Abstract

Osteosarcoma is a malignant tumor derived from primitive osteogenic mesenchymal cells, which is extremely harmful to the human body and has a high mortality rate. Early diagnosis and treatment of this disease is necessary to improve the survival rate of patients, and MRI is an effective tool for detecting osteosarcoma. However, due to the complex structure and variable location of osteosarcoma, cancer cells are highly heterogeneous and prone to aggregation and overlap, making it easy for doctors to inaccurately predict the area of the lesion. In addition, in developing countries lacking professional medical systems, doctors need to examine mass of osteosarcoma MRI images of patients, which is time-consuming and inefficient, and may result in misjudgment and omission. For the sake of reducing labor cost and improve detection efficiency, this paper proposes an Attention Condenser-based MRI image segmentation system for osteosarcoma (OMSAS), which can help physicians quickly locate the lesion area and achieve accurate segmentation of the osteosarcoma tumor region. Using the idea of AttendSeg, we constructed an Attention Condenser-based residual structure network (ACRNet), which greatly reduces the complexity of the structure and enables smaller hardware requirements while ensuring the accuracy of image segmentation. The model was tested on more than 4000 samples from two hospitals in China. The experimental results demonstrate that our model has higher efficiency, higher accuracy and lighter structure for osteosarcoma MRI image segmentation compared to other existing models.

## 1. Introduction

Osteosarcoma is one of the most common bone malignancies, which develops from mesenchymal cell line [1]. Although it accounts for 0.2% of human malignant solid tumors, the mortality and disability rates are very high. Osteosarcoma is the most common type of bone malignancy in the elderly and children, and the second peak of its morbidity is over 60 years old [2,3,4]. At present, the five-year survival rate of osteosarcoma patients in China is about 60%, and the ten-year survival rate decreases to only 30%. Moreover, during the five-year or ten-year survival process, most patients still need to experience metastasis, recurrence, multiple operations, etc. [5,6,7,8]. The size, position, architecture, and shape of osteosarcomas will be diverse depending on the patient’s physiology, physical condition, and degree of disease. Additionally, the distribution density of osteosarcoma is not uniform. It is often difficult to distinguish the tumor tissue from the surrounding normal tissue [9,10,11,12,13].

The diversity of osteosarcoma leads to scattered information about it in the medical literature, particularly in imaging results. The images of diverse osteosarcoma generated in the identical environment and process are various as well, and it is hard to tell the difference between healthy tissue and lesion areas manually in some cases [14,15]. Generally, the examination of osteosarcoma is nothing more than X-ray examination, CT scan, and MRI examination. Among the three methods, magnetic resonance owns great soft tissue identification as well as an exceptionally high Contrast Ratio, and its ability to slice in multiple parameters and planes allows for clear visualization of the position and degree of the lesion, in the meantime, the harm to the patient’s body during the detection is also the smallest [16,17].

The mortality of osteosarcoma is very high, but early detection and timely treatment can greatly improve the survival rate [18]. However, the imaging diagnosis and treatment of osteosarcoma has faced barriers due to the poorly developed medical infrastructure in most of developing countries. The need for early diagnosis of osteosarcoma is made difficult by the high price of MRI machines and the lack of top-level talent. To make matters worse, the diagnosis of a single patient will generate 600-700 MRI images. Of the vast quantity of data, frequently less than 20 of them are considered usable [19]. The rest of the redundant images not only slow down the progress of judgment, but also sometimes mislead doctors to a certain extent [20]. In addition, considering that the diagnosis of MRI images is highly subjective and mostly depends on the experience and professional knowledge of doctors, the lack of talents in this field is also a major reason for the low efficiency of diagnoses [21]. The low recognition rate of early diagnosis leads to more difficult later-stage treatment, forming a vicious circle [22,23]. In China, 80% of medical resources are concentrated in large cities with only 10% of the population. In remote areas and some underdeveloped small towns, the lack of medical resources has led to a serious imbalance in the doctor-patient ratio, leaving people with only simple medical care. This series of problems is heavy grief for families and societies [24,25,26]. It is obvious that if we want to promote the medical system in this area more quickly and comprehensively, we need to realize low-cost, efficient, and accurate image segmentation technology to replace the original diagnosis methods, and use machines for initial screening to provide diagnostic assistance for doctors [27,28].

In recent years, with the increasing attention to AI, some medical image detection technologies have also been applied to the diagnosis of osteosarcoma [29,30]. Although these techniques can to some extent detect the position and margins of the tumor, the accuracy of detection is not high due to the variability of osteosarcoma [31,32,33,34]. The effect of existing medical image diagnosis technology in osteosarcoma image segmentation is not as expected [35]. The methods of machine learning are to manually calibrate the original data and then establish the mapping between the image and the calibrated area through a function. Then, the parameters of the function will be trained by using a large amount of data so that the function can extract some information between them that we cannot extract [36,37,38]. Previous models often used elaborate architectures as well as deeper hierarchies to improve their fitting ability and thus give them higher accuracy. While this approach can increase the model’s accuracy to some extent, it tends to reduce the generalization ability of the model, and thus the effect of the improvement is on the weak side often [39,40]. Further, excessively sophisticated architecture will lead to slow and less efficient model training, and the hardware demands will likely be high.

According to the above contents, we propose an MRI image segmentation and AI-assisted diagnosis system based on Attention Condenser for osteosarcoma (OMSAS) to assist doctors. This intelligent medical system is designed to assist doctors in identifying MRI images of patients with osteosarcoma and to automatically segment osteosarcoma, thus providing doctors with more powerful and intuitive tips and aids. The analysis results provided by the system can be used as an auxiliary basis for doctors to diagnose patients, which can effectively improve the efficiency and accuracy of diagnosis and reduce the cost of diagnosis and treatment for patients. Firstly, we carry out data augmentation in the dataset and use a variety of methods to expand, standardize and classify the original dataset. Data preprocessing reduces the degree of over-fitting and makes the model more generalized. In terms of model design, we improve AttendSeg [41], replace the ordinary convolution layer with two blocks we designed and delete several unnecessary layers. We designed a residual network based on Attention Condenser (ACRNet), which uses a residual network structure and reconstructs the most important component Attention Condenser [42] in AttendSeg. Compared with other models combining the residual structure, ACRNet retains the self-attention mechanism in Attention Condenser that learns and generates cohesive embeddings characterizing joint local and cross-channel activation relationships, further enhancing the model’s attention to osteosarcoma regions. The self-attention mechanism enables the model to migrate more attention to refine the details between regions in the osteosarcoma MRI image, better extract various features of the image, and ultimately improve the accuracy of segmentation. In addition, the ACRNet after component reconstruction facilitates for sparser use of larger stand-alone convolution modules to reduce overall network complexity and has a lower number of parameters. Finally, in terms of output results, we choose to change a single output into a composite decision, calculate its average results and generate a binary map to facilitate the physician’s diagnosis.

The specific contributions of the essay are divided into the following points:

(1) In this article, the region in the image can be focused more accurately through the Attention Condenser. The multilayer condenser structure can further locate the boundary of the tumor, and reduce a large number of unnecessary calculations in the later stage, to improve the efficiency and accuracy of training.
(2) When using the model to predict, OMSAS uses a compound decision module that turns a single input into multiple copies of the input. Then, the model makes predictions by multiple copies simultaneously, and then combines the multiple outputs to get the final result. Multiple decisions can reduce the wrong prediction caused by some unknown situations and make the output more accurate. Meanwhile, it can improve the stability of the model.
(3) More than 4000 images provided by the First People’s Hospital of Huaihua and the Second People’s Hospital of Huaihua were used for testing. The result indicates that our ACRNet in OMSAS is better than other existing segmentation models. The model has high training efficiency and prediction accuracy as well as small resource consumption, which is important in assisting doctors to diagnose osteosarcoma in patients.

The paper is arranged as follows: Section 2 introduces some research related to our work. In Section 3, we depict the main structure and design of the Attention Condenser-based MRI image segmentation system for osteosarcoma (OMSAS). Section 4 presents the experimental dataset and shows the performance of the model through evaluation metrics to demonstrate the validity of the method. At the end of the paper, we summarize our work and look forward to future work.

## 2. Related Works

Through investigation and research, we find that many technologies use artificial intelligence knowledge for medical decision-making and image processing. In modern medical systems, more and more artificial intelligence (AI) algorithms are used for image segmentation, health prediction, and other functions. In the diagnosis of osteosarcoma, how to process MRI images and accurately mark the tumor area has become a research hotspot. This section will introduce some mainstream algorithms in related directions.

Nasor et al. [43] proposed an image processing technique in their study to classify osteosarcoma into different MRI scan types using techniques such as K-means, Chan-Vese segmentation, etc. This method can reduce the impact between different MRI images by first classifying and then detecting, and can more accurately predict the regions of various images in advance. Kayal et al. [44] used nine segmentation algorithms (OT, OT-RG, AC, SLIC-S, FCM, GC, LR, L-SVM and DNN) to segment DWI and achieved good results. Whether semi-automatic like OT-RG or full-automatic like LR, it can reduce the workload of doctors to a certain extent. Nabid et al. [45] used Sequential Regions with CNN features (RCNN) to segment osteosarcoma images. RCNN using multiple CNN blocks synthesized by Gated Recurrent Units and dense networks has better output results than traditional CNN models such as VGG16 and ResNet-50.

Arunachalam et al. [46] used support vector machine (SVM) and other machine learning models to estimate the pathology of tumor necrosis after chemotherapy. At the tissue and cell level, different regions of the digitized picture are marked as viable tumor, necrotic tumor, and non-tumor, helping some pathologists better identify the corresponding features. Additionally, they employed K-Means Clustering technology to separate tumors by color normalization at the cellular level. Then, with the support of composite threshold-Otsu segmentation technology, the tumor region is further divided into viable and non-viable.

Dionisio f.c.f et al. [47] took Hausdorff distance (HD) and dice similarity coefficient (DSC) as the main research objects in the study and compared the artificial segmentation results with the machine segmentation results. They pointed out that DSC was considered satisfactory between 0.61 and 0.80, and almost perfect or excellent between 0.81 and 1.00. The study shows that the average DSC of manual segmentation is 0.91, and the average reading time is about 616.8 ± 390.1 s. The F-HHO model proposed by Badashah et al. [48] is a generative adversarial network (GAN) based on the Fractional-Harris Hawks optimizer, which performs the detection of osteosarcoma by extracting characteristics from the images during the cell image segmentation process. F-HHO has reached more than 95% in accuracy, sensitivity, and specificity. Anisuzzaman et al. [49] conducted training on whole slide images (WSI) to test the transfer learning model containing VGG19 and Inception-V3. Finally, the results show that VGG19 is the best in the tested model, with an accuracy of 96%.

In the field of image segmentation, there are many good models in recent years, which can be divided into threshold-based, cluster-based, edge-based, region-based, etc [50]. U-Net proposed by Ronneberger et al. [51] uses skip connection and depth monitoring to achieve a good segmentation. Full-scale skip join contains high-level semantics with low-level details in feature maps from different scales, and depth monitoring learns the hierarchical representation from the full-scale aggregation feature map. Song et al. [52] compared the threshold-based segmentation methods and found that the Ostu method was used to divide the picture into many small blocks, and then determined the local threshold of each small piece, which can be well segmented in the case of uneven illumination and blurred image. Gao et al. [53] proposed an image segmentation strategy based on band conversion, making detailed segmentation in horizontal, vertical, and diagonal by using wavelet transform theory. This method has a better effect than traditional variance segmentation and dual-mode segmentation, especially in medical image segmentation. Fang et al. [54] constructed the segmented pipeline using the joint adversarial and segmentation network and proposed a segmentation model called SUSAN, which reached the same level as the supervised U-Net in knee image segmentation. MSFCN proposed by Huang et al. [55] is a fully convolutional neural network based on multi supervision. Its upsampling part further improves the segmentation accuracy by using the composite feature extraction channel to capture more context information. In the research of Zhang et al. [56], the proposed model MSRN is a multiple supervised residual network. Adding three monitoring side output modules to the network can not only extract the shape features of the image, but also extract the semantic features. By fusing the results of three side output modules, the final segmentation result is obtained. The feature pyramid networks (FPN) designed by Lin et al. [57] in this direction use the multi-scale pyramid hierarchy of deep convolution network to construct the feature pyramid at the boundary additional cost. FPN develops a top-down architecture with horizontal connections, which can be used to build high dimension semantic feature maps at all scales. Shelhamer et al. [58] defined a jumping architecture through the fully convolutional network with 8 times upsampling (FCN-8s) to classify images at the pixel level. The input of any size can be accepted, and the deconvolution layer is used to upsample the characteristic image of the last convolution layer.

Through the above research, we found that with the continuous development of computer technology, the research scope of artificial intelligence technology in the field of paramedicine, especially in the direction of image recognition, is expanding. However, due to the variability of the morphology and structure of osteosarcoma, existing medical image recognition techniques are difficult to achieve the expected results in MRI image segmentation of osteosarcoma. To improve the segmentation accuracy and better adapt to some medical devices with relatively poor performance, we design a new strategy for MRI image segmentation for osteosarcoma based on Attention Condenser. The approach enhances the efficiency and accuracy of osteosarcoma detection by reducing the device requirements via tactics such as data preprocessing, residual network, LayerNorm and attention condenser.

Considering that we have submitted two similar papers [59,60], the following explains the difference and novelty between our paper and these two papers.

We have conducted a comparative analysis of the innovations in the three papers. First, all three papers address the fact that osteosarcoma is extremely dangerous for human beings and that the diagnosis and treatment of osteosarcoma in developing countries is extremely difficult due to the shortage of medical resources. Moreover, there are few specialized physicians due to the large volume and complexity of patient data. Therefore, we designed an osteosarcoma artificial intelligence method to assist physicians in picture analysis and clinical diagnosis.

However, the three studies have different emphases, and there are differences in the problems they each address.

For literature [59], it uses osteosarcoma histopathological images as a research object to achieve classification of abnormal pathological images. Its main purpose is to address the sensitivity of medical datasets and the scarcity of labeled data bringing limitations to the performance of artificial intelligence methods. The method effectively improves the labeling gain of osteosarcoma pathology images by actively acquiring the most characteristic pathology images as labeled samples.

For literature [60], this method uses MRI images of osteosarcoma as the object of study and performs segmentation of osteosarcoma by real-time segmentation network. The main purpose of this method is to improve the accuracy of the system segmentation by removing noise through the pre-Eformer model and localizing and enhancing the tumor region using nonparametric localization and enhancement methods to make the osteosarcoma appear more clearly shaped. However, the system requires a lot of time to process the images during the pre-processing process, increasing the cost of model training. Although this segmentation network can achieve accurate segmentation of multi-scale tumors, the model is prone to receive limitations from external features. The MIR images are from different devices, and the sensitivity of the images varies and may be contaminated due to the environment, equipment, and operators.

Compared with the above two papers, our paper uses a more novel MRI image segmentation model for osteosarcoma, ACRNet. The ACRNet model is more novel, and the model training is more efficient and achieves better segmentation results. This is reflected in the following points.

1. In terms of data preprocessing, we use a simpler and more efficient processing method that maintains a good segmentation performance. We binarize and regularize the images to filter out the valid regions in the images and eliminate the effect of different brightness levels between images on the training. Additionally, we enhance the dataset to improve the generalization of the model.
2. In terms of model design, we refactored the main component of AttendSeg, “Attention Condenser”. We use the attention mechanism and combine it with the residual structure to further enhance the attention of the model to the osteosarcoma region, so that the model can shift more attention to refine the details between regions in the osteosarcoma MRI image, and understand the global view of the image, correct the results in the reconstruction, and effectively improve the segmentation effect. In addition, we transformed the original general convolutional layer combination into a reserved block and a shrinkage block, which greatly reduces the number of parameters of the general convolutional operation and allows the model to better extract various features of the image and run more efficiently.
3. In the output part of the model, we use the composite decision to integrate the output of multiple different angles of the same image and unify the results of the same source, which effectively enhances the accuracy and robustness of the output results. We plot the output results as a black-and-white image to assist the doctor’s diagnosis, thus greatly reducing the burden of the doctor’s film reading.
4. In terms of the practicality of the segmentation system, the network has a lightweight structure with only 6.91 M parameters and a SETT value of 174, which makes the model simpler and more efficient in training and more adaptable to low-configuration medical equipment, which helps the implementation of the medical-assisted segmentation system on the ground. As a result, ACRNet ensures high accuracy in segmenting osteosarcoma MRI images while improving efficiency, which will save more human and financial resources for developing countries and improve the efficiency of osteosarcoma diagnosis in hospitals.

## 3. System Model Design

This section may be divided by subheadings. It should provide a concise and precise description of the experimental results, their interpretation, as well as the experimental conclusions that can be drawn.

At this stage, in most developing countries, the distribution of medical resources is very unbalanced, and many advanced medical resources are concentrated in the region where very few people are located. Additionally, generally speaking, a particularly good analytical instrument can greatly reduce the working pressure of doctors. However, the high manufacturing and maintenance costs make it very difficult to promote the medical equipment of image-assisted diagnosis.

**Figure 1:**
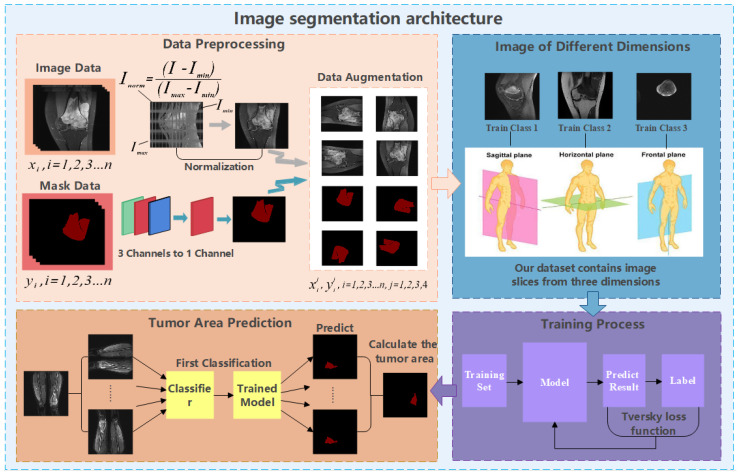
Architecture diagram of OMSAS.

Considering the osteosarcoma patients living in developing countries, if the early diagnosis is not timely, the lack of technology or the very high cost of later medical treatment will often make them give up their last chance of survival. Therefore, what we need to do is to improve the early diagnosis rate. For hospitals, the prolonged manual diagnosis and the shortage of talents result in the difficulty of diagnosis. It is necessary to introduce an osteosarcoma MRI image segmentation medical system to assist doctor. Moreover, to promote this system more widely, we expect that the built-in process should be simple and efficient enough, the model should have high accuracy, and the requirements for equipment should be as low as possible. Thus, based on these cognitions, this paper proposes a segmentation method for osteosarcoma MRI image based on Attention Condenser (OMSAS), which can accurately depict the tumor area in the image, relieving the burden of reading films for doctors. The overall design of the system is shown in Figure 1.

According to Figure 1, we can see that the system we proposed in this paper is roughly divided into three parts, including data preprocessing, model training, and model application. This chapter will be divided into four sections. In Section 3.1, we introduce the work of data preprocessing. In Section 3.2, we briefly introduce the attention mechanism. Section 3.3 and Section 3.4 elaborate the ACRNet segmentation model proposed in the paper and the loss function, respectively.

Some symbols involved in the chapter are explained, as shown in Table 1.

### 3.1. Data Preprocessing

We find that the data in the original dataset is not suitable for model training directly, and there are the following problems:

(1) The osteosarcoma MRI images in the dataset have different brightness and darkness due to different instruments, contrast agent dose, and other external environmental reasons;
(2) The amount of data is still insufficient for training a model with high accuracy, and the training is prone to instability.

**Figure 2:**
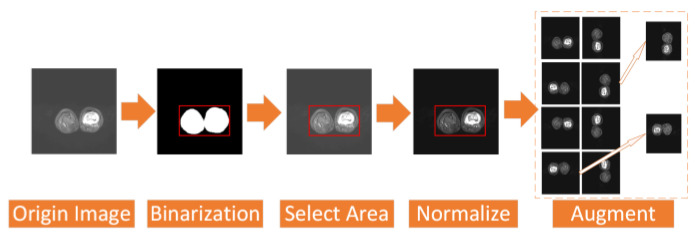
Process of data preprocess.

To address the above two problems, we have carried out a series of data preprocessing to optimize the effect of the model by improving the quality of data and eliminating interference factors. The specific operation is shown in Figure 2.

First, we select the region of all the osteosarcoma MRI images. After the image is binarized by (1), it will be filled to obtain a picture with only pure black and pure white areas. Then, we select the smallest rectangular area in the picture that can just contain the pure white area, which is the selected effective area. Selecting the effective region can simplify the training and recognition of the model, and make the model pay more attention to the feature extraction of the effective region.

(1) $$p_{binary}=\frac{1}{2}+\frac{1}{2}\times \frac{p-\mu_{pixel}}{\left|p-\mu_{pixel}\right|}$$

In addition, we regularize all the osteosarcoma MRI images to avoid the impact of some excessively bright and dim images on the training. The brightness gap and different density distribution between images make it easy for the model to take these unnecessary factors into account, which not only reduces the speed of model training, but also may affect the effect of the model. Therefore, we use a unified regularization process to transform all MRI images with (2).

(2) $$p_{norm}=\frac{p-p_{min}}{pmin_{max}}$$

The regularized image addresses the first problem. At this time, the image has met the minimum standard of training. However, to enhance the robustness of our model and the generalization of it, we also need data augmentation. For data augmentation, we rotate each picture by 90°, 180°, and 270° at first, and then flip each picture horizontally and vertically. After that, the amount of data is 8 times that of the original dataset. We then randomly select some images and add noise to them to reduce the overfitting phenomenon of the model when learning high-frequency features.

After data preprocessing, the quality of the MRI images of osteosarcoma in the dataset was improved, and the high-quality dataset can be used as a reference basis for clinical diagnosis, which is beneficial for doctors to make more effective film reading. At the same time, the enhanced dataset can provide a good fit for the model training.3.2. Brief Introduction of Attention.

### 3.2. Brief Introduction of Attention

The model of this paper involves the mechanism of attention, so attention will be briefly introduced in this part.

We know that the inspiration of the attention mechanism comes from our physiological perception of the environment. Our visual system will actively select the information we need to pay attention to in the impression and ignore the irrelevant information in the field of vision. Similarly, in the neural network, when we segment an osteosarcoma MRI image or recognize the boundary, we also hope the network to pay more attention to the region we expect, and ignore the irrelevant information. The attention mechanism can be divided into three steps: compression, activation, and attention. For example, we now have an osteosarcoma MRI image input *M*. and the size of *M* is (*w*, *h*, *c*). Moreover, *w* means width, h represents its height, and c is its number of channels. Then, the compression process can be expressed as (3).

(3) $$z_{c}=\frac{1}{ℎ\times w}\overset{w}{\underset{i=1}{\sum }}\overset{ℎ}{\underset{j=1}{\sum }}M_{c,i,j}$$

In order to evaluate the importance of each channel, we need to activate the previous operation results as a whole. The weight calculation formula on each channel is shown in (4), where $W_{\rho }$ and $W_{\sigma }$ are two weight matrices. After their respective matrix operations, they will be input into σ. The ρ representing the sigmoid function and the ReLU function.

(4) $$e_{c}=\rho \left(W_{\rho }\sigma \left(W_{\sigma }z_{c}\right)\right)$$

After the activation, we assign M to the weight matrix $e_{c}$ corresponding to the input M. After $e_{c}$ is multiplied by M, we get $M^{\prime }$ after adjusting the channel weight. It is written as (5).

(5) $$M^{\prime }=M\cdot e_{c}$$

**Figure 3:**
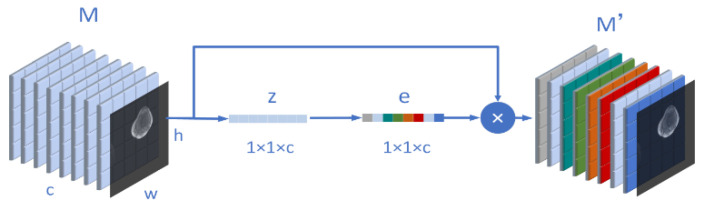
Schematic diagram of the structure of the attention mechanism involved in this paper.

In our model design, attention can be understood as the process of Figure 3. Through this mechanism, the algorithm can pay more attention to useful information, ignore many useless parts, and reduce the amount and complexity of calculation.

### 3.3. Osteosarcoma MRI Image Segmentation Model

**Figure 4:**
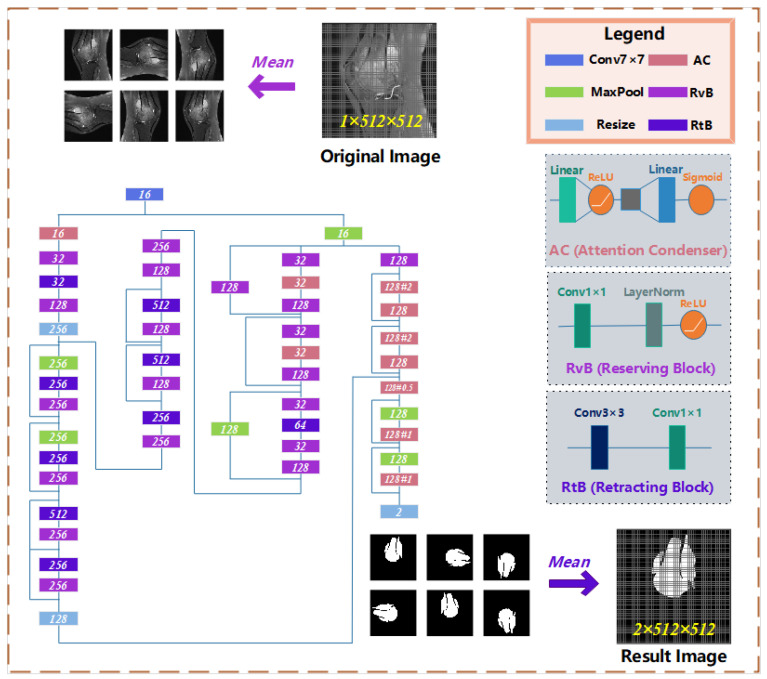
The architecture of osteosarcoma segmentation network ACRNet.

On the osteosarcoma image segmentation model, we designed a residual network based on Attention Condenser (ACRNet), and its overall structure is shown in Figure 4. The whole network is split into three main parts as follows:

(1) The osteosarcoma MRI images will be rotated and flipped to obtain a group of pictures before each prediction of it.
(2) The set of pictures from the previous step will be used as input for the residual network with multiple attention condensers, and a set of results will be output.
(3) This group of results is inversely changed according to the original change direction to obtain a group of results consistent with the original image direction. This group of results is calculated and processed to obtain the final prediction tumor region.

In recent years, a large number of scholars have emerged to conduct research on attention networks. The attention condenser proposed by Alexander Wong et al. [42] uses the self-attention mechanism to reduce the size of the model and does not affect the performance. Attention Condenser is designed to capture spatial channel activation relationships by learning the aggregation embedding of activation relationships to achieve efficient selective attention. ACRNet incorporates a residual network with multiple Attention Condenser, highlighting the superiority of the structure. On the one hand, Attention Condenser, as an independent stand-alone module, jointly models both local and cross-channel activation relations within a unified condensed embedding using a self-concentration mechanism, which facilitates for sparser use of larger stand-alone convolution modules to reduce overall computational complexity of such joint modeling and unnecessary computations in the ACRNet. On the other hand, the multilayer condenser-based ACRNet can localize the boundaries of tumors and focus more accurately on the osteosarcoma region in MRI images, which further improves the accuracy of image segmentation.

Attention Condenser consists of four parts: a condensed layer that reduces the dimension of spatial channels, an embedded structure that represents the activation relationship of joint spatial channels, an extended layer that increases the dimension, and an attention mechanism that applies selective attention. In ACRNet, we use two linear structures and the middle embedding layer to perform the above scaling operation. In addition, we introduce a variable called ratio, which can manually adjust the expansion and contraction times of the condenser, to meet the needs in different situations better.

The residual structure is used many times in the whole network. The residual unit is realized in the form of layer skip connection. Some layers are regarded as a unit. The initial input of the unit and the final output will be added before activation. The residual structure addresses the degenerate problem of the deep neural network. Under the same number of layers, the training speed of the residual network is faster, which can make the operation efficiency of the model higher.

In order to better extract various features of the osteosarcoma MRI image, we use the idea of the block to combine and transform the original general convolution layer into the reservation block and the retracting block. The reservation block includes a 1 × 1 convolution layer, a Layer Normalization, and a ReLU activation function, which only changes the number of channels of input without affecting the size of the image. Adding LayerNorm can normalize the data before entering the activation function to avoid the problem of vanishing gradient in the saturated region of the activation function. Meanwhile, the use of LayerNorm accelerates the convergence speed of the model again.

The retracting block consists of a 3 × 3 and a 1 × 1 convolution layer. Its purpose is to simply extract image features. A retracting block instead of single 3 × 3 convolution layer makes a convolution kernel responsible for only one channel. A channel will be convoluted by only one convolution kernel, and the change of channel is handed over to 1×1 convolution layer. This can significantly reduce the parameters number of the ordinary convolution operation.

In the output, we use compound decision-making to integrate multiple outputs from different angles of the same image, since that for neural networks, every rotated or flipped image is a new image, and the prediction results are not necessarily the same. Unified processing of homologous output can enhance the accuracy and robustness of results. The specific formula for output calculation is as follows.

(6) $$r_{j,k}=\frac{1}{2}-\frac{1}{2}\times \frac{\sigma_{j,k}-\frac{n}{2}}{\left|\sigma_{j,k}-\frac{n}{2}\right|},\sigma_{j,k}=\sum_{i=0}^{n}p_{i,j,k}(j=0,1,2,\ldots ,h;k=0,1,2,\ldots ,w)$$

### 3.4. Loss Function

Medical image segmentation models often encounter the phenomenon of data imbalance. Training unbalanced data is easy to cause the problem of high precision but low recall. Moreover, in the diagnosis of osteosarcoma, missed diagnosis of tumor area is a more intolerable serious error than the wrong diagnosis of the normal area. Therefore, when designing the model, we choose Tversky Loss as the loss function. Tversky loss is a special dice loss, which is a combination of dice loss and Jaccard coefficient. Generally, the weight of dice loss for FP and FN is equal, so the model will not focus on the improvement of recall Sufficiently during training. Therefore, we use Tversky Loss with parameters β as the loss function in model training. The specific definition is as shown in (7).

(7) $$T(\alpha ,\beta )=\frac{\sum_{i=0}^{N}{\mathbb{R}}_{i}{\mathbb{Q}}_{i}}{\sum_{i=0}^{N}{\mathbb{R}}_{i}{\mathbb{Q}}_{i}+\alpha \sum_{i=1}^{N}{\mathbb{R}}_{i}(1-{\mathbb{Q}}_{i})+\beta \sum_{i=1}^{N}{\mathbb{Q}}_{i}(1-{\mathbb{R}}_{i})+\varepsilon }$$

*α* and *β* control the penalty strength of the loss function for FP and FN, respectively. The higher the β, the greater the penalty of the loss function on FN, and the easier it is to improve the recall. After testing, we adopt *α* = 0.25, *β* = 0.75.

By using our model, we can easily perform region detection on osteosarcoma MRI images and the annotation regions are comparable to the “Gold Standard” of doctor annotation. Our model ensures accuracy while greatly reducing the burden on doctors when reading MRI images. For hospitals, the time and money spent on processing related cases are greatly reduced.

## 4. Performance Evaluation

### 4.1. Dataset Introduction

The data in the article are collected from the First People’s Hospital of Huaihua and the Second People’s Hospital of Huaihua. During the experiment, we collected more than 4000 MRI images and other related index data of 210 patients with different degrees of osteosarcoma. During the experiments, all segmentation models are built on the same dataset, so the performance tests of the models are comparable. To ensure that the sample is closer to the standard of developing countries, we selected data mostly from patients with not very high income in the lower social class. Additionally, the data are mostly from early imaging and a small percentage from mid to late. We selected three different cross-sectional slices to allow the model to better fit multiple input cases and to achieve stable segmentation results. All MRI images are selected and manually labeled by doctors. We divided the dataset into the training set and the test set in the ratio of 7:3. Of the 210 real cases of patients we owned, 147 are put into training set and 63 are in test set. We used a 10-fold cross-validation to ensure the validity of experimental results.

The training, test environment, and some important parameters settings during the training of the model in this paper are shown in Table 2.

### 4.2. Detection Index

In the process of training and testing, better, we will use some common comparative index and single epoch training time (SETT) as metrics to evaluate the performance of our ACRNet model and other segmentation models. Moreover, we will also make a comparison of the number of parameters of the different models.

We used the confusion matrix to analyze and explain the model. TP indicates that both the predicted results and the actual results are osteosarcoma areas, and TN indicates that both the predicted results and the actual results are normal areas. FP represents the normal area misjudged as the disease area, and FN represents the lesion area misjudged as the normal area. The specific definition and calculation formula of the above indicators are as follows.

**Definition** **1.** *Accuracy. It represents the proportion of all samples with correct prediction. Its definition is as shown below.*

(8) $$Accuracy=\frac{TP+TN}{TP+TN+FP+FN}$$

**Definition** **2.** *Precision. It represents the proportion of all samples judged as tumor areas that are actual tumor areas. The formula for Precision is:*

(9) $$Precision=\frac{TP}{TP+FP}$$

**Definition** **3.** *Recall. It represents the percentage of all samples judged as normal areas that are really normal areas. Compared with precision, we expect the emergence of high recall in medical experiments, and false negative is more intolerable than false positive. Its definition is as follows.*

(10) $$Recall=\frac{TN}{TN+FN}$$

**Definition** **4.** *F1-Score. It is an index based on precision and recall, which can represent the robustness of a model. The higher the F1-Score, the greater the robustness. Its definition is as shown below.*

(11) $$F1=2\times \frac{Precision\times Recall}{Precision+Recall}$$

**Definition** **5.** *IOU. It represents the intersection and union ratio between the tumor region in the prediction result and the tumor region in the real result, reflecting the coincidence between the prediction result and the real region. Its definition formula is as follows*, where $I_{pred}$* represents the tumor region in the predicted result and *$I_{real}$* represents the tumor region in the real result.*

(12) $$IOU=\frac{I_{pred}\cap I_{real}}{I_{pred}\cup I_{real}}$$

**Definition** **6.** *DSC. It describes the similarity between the prediction result and the truth and is an index with a value of 0-1. The closer DSC is to 1, the closer the result is to the real situation. Its definition formula is as follows.*

(13) $$DSC=2\times \frac{\left|I_{pred}\cap I_{real}\right|}{\left|I_{pred}\right|+\left|I_{real}\right|}$$

**Definition** **7.** *SETT. It represents the average time required for an epoch of model training under the same amount of data, and the unit is seconds (s).*

### 4.3. Comparison Model

For the purpose of further evaluating the segmentation effectiveness as well as the structural complexity of our ACRNet, we used U-Net [51], MSFCN [55], MSRN [56], FPN [57] and FCN-8s [58] to compare various metrics with the ACRNet model, which have achieved some achievements in image segmentation. In the related work, we have made certain presentations.

### 4.4. Operation Results and Analysis

**Figure 5:**
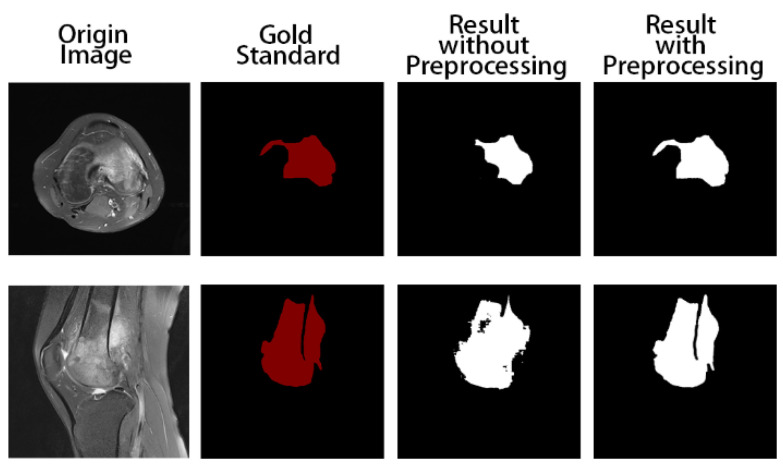
Comparison of segmentation results of the model with and without data preprocessing.

Firstly, we trained the model without preprocessing. The training and test results are shown in Figure 5. The tumor areas marked in red are manually marked by professional doctors. In the two pictures marked in white, the one on the left is the result of model prediction obtained by training without preprocessing, and the one on the right is the result of model prediction obtained by using the preprocessed data as input. We can obviously find that the segmentation results with the preprocess are at closer proximity to the gold standard, while the output of the model without data preprocessing is not satisfactory. In addition, it can be seen that ACRNet with data preprocessing achieves better segmentation results for images with obscure osteosarcoma boundaries. Therefore, ACRNet makes the model more stable by using the strategy of compound decision to integrate multiple outputs from different angles of the same image, which effectively improves the segmentation effect of the model for the osteosarcoma region.

**Figure 6:**
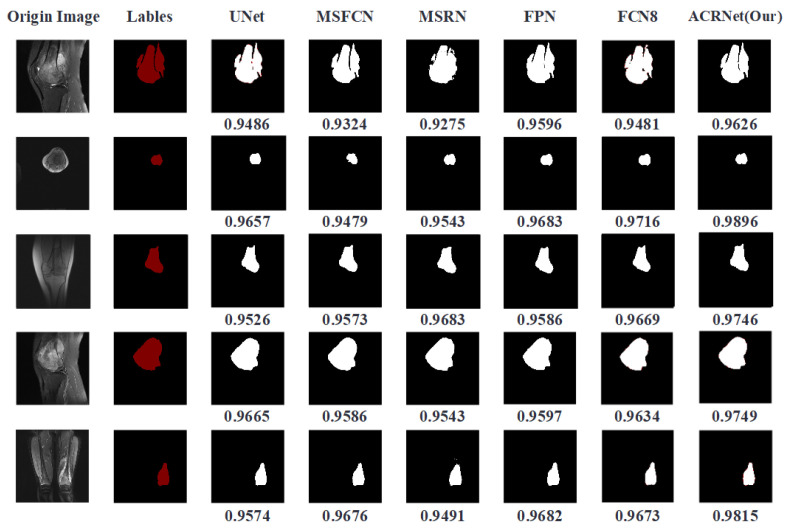
Comparison of segmentation results of each model on different images.

We trained the model and finally got a trained ACRNet model. Then, we put the prepared test data into it for testing and test it synchronously with other prepared image segmentation models. The tumor region prediction images are shown in Figure 6. The first column represents the original MRI image, which contains three different sections from different patients. The second column is the tumor area manually marked by doctors, which is called “Gold Standard”. Columns 3 to 7 are the predicted results of previous models we choose to compare. The last column is the tumor area map predicted by ACRNet. The DSC metrics of the predicted results of the MRI image under different models are recorded at the bottom of each line, and the highest index is marked in bold form.

According to Figure 6, we can find that in the five selected pictures, ACRNet shows good performance and the DSC metrics are higher than those of the other models. Especially in the image segmentation from the perspective of transverse, the result of ACRNet has obvious advantages, which is about 4 percentage points higher than that of the U-Net model. Based on these five examples of osteosarcoma segmentation, we can draw the conclusion that ACRNet can segment the osteosarcoma images with better accuracy and can be consistent with the manual annotation called Golden Standard.

To compare each model more accurately and digitally, we counted the segmentation results of each model and calculated and sorted out its indicators. We used several indicators selected and defined in section C to compare our model with other models. Table 3 shows different indicators of prediction results of different models under the same data set and the same operating environment. As shown in Table 3, we found that ACRNet showed a good effect on MRI image segmentation of osteosarcoma, and achieved higher values than others in most index comparisons, like IOU, recall, etc. These indicators are relatively high, which indicates that the model has better generalization and robustness, and can identify segmentation more accurately. To compare the computational complexity of the models, on the one hand, we compared the SETT metrics of ACRNet with those of UNet, MSFCN, MSRN, FCN and FCN-8s models. The SETT of ACRNet is only 174s, which is significantly lower than other segmentation models, indicating that it has higher computational efficiency than other models. On the other hand, the number of parameters of ACRNet is also the lowest among all models, only 6.91 M. This shows that ACRNet has lower computational complexity than other models. The low computational complexity can better accommodate medical devices with low configuration (e.g., CPU, GPU, etc.), which undoubtedly saves more human and financial resources for developing countries and can improve the efficiency of diagnosis of osteosarcoma in hospitals.

**Figure 7:**
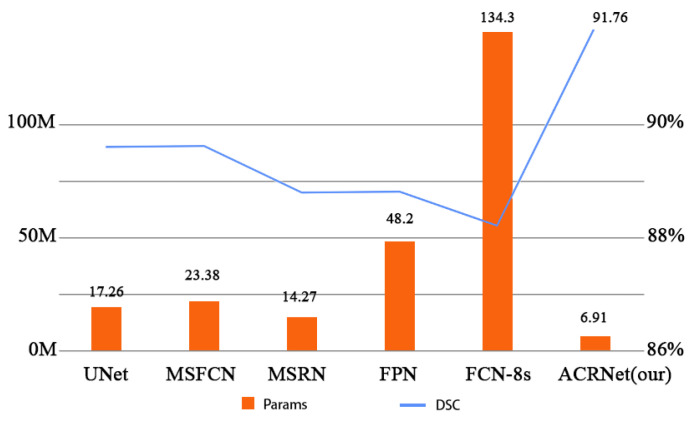
Comparison of Params and DSC between different models.

Figure 7 more intuitively and specifically shows the comparison of parameters between different models and the changes of DSC. In Figure 7, the horizontal coordinates represent the different models, and the vertical coordinates are divided into two indicators on both the left and right. The left vertical coordinate indicates the quantity of parameters of these models, and the right vertical coordinate indicates the DSC metric of the model. The chart shows that ACRNet has lower number of parameters than UNet, MSFCN, MSRN, FCN, and FCN-8s models, with only 6.91 M parameters, which is nearly 7.4 M parameters lower than the MSRN model. Meanwhile, ACRNet is not inferior in osteosarcoma image segmentation because of the small number of parameters, with a DSC of 91.76%. Compared with the MSFCN model, which ranked second with a DSC of 89.2%, the DSC of ACRNet was nearly 2.5% higher. This result further shows that our model will take up less memory, and the cost of each training can be much lower than other models, which can save more medical expenses for some underdeveloped areas.

**Figure 8:**
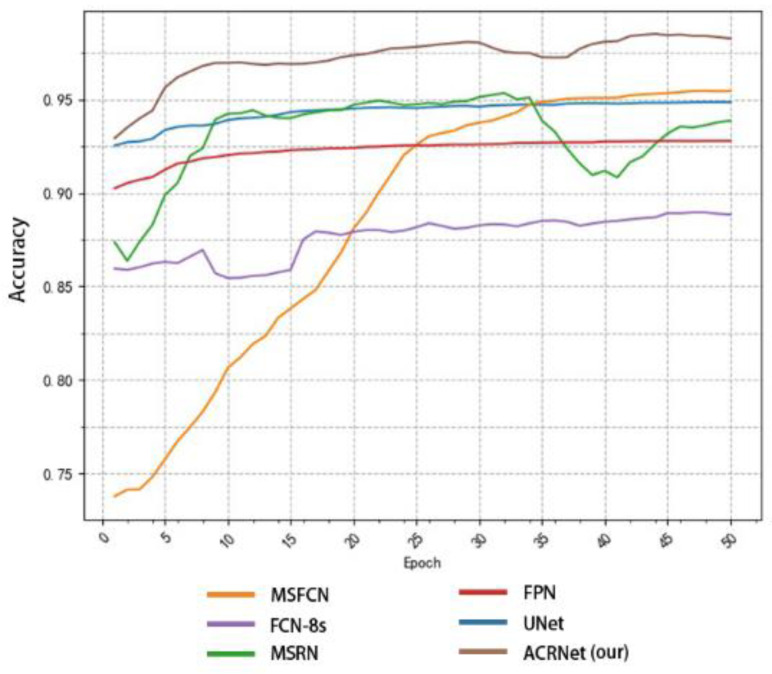
Variation of the accuracy of each model with the number of training epoch.

To achieve a better prediction effect, we often need to train more epochs to make the model fit the dataset better. We often expect a good model to converge to a higher accuracy as soon as possible, and the higher the final convergence value, the better. Based on this, we tested different models as shown in Figure 8. We selected some of the training records of the first 50 epochs from the log file for drawing a line diagram. According to Figure 8, we find that the initial accuracy of most models can reach more than 80%, and ACRNet reaches 88%. With the increase of training epochs, ACRNet began to converge rapidly and reached more than 95% accuracy in the early stage. Compared with the rapid rise of MSFCN and the stable convergence of U-Net, ACRNet not only remains stable after convergence but also the accuracy maintains at a high level. Generally speaking, ACRNet has good convergence efficiency and effects, which means that the model can obtain a good prediction result with fewer training epochs and less training time than other models. It can improve diagnostic efficiency for doctors.

**Figure 9:**
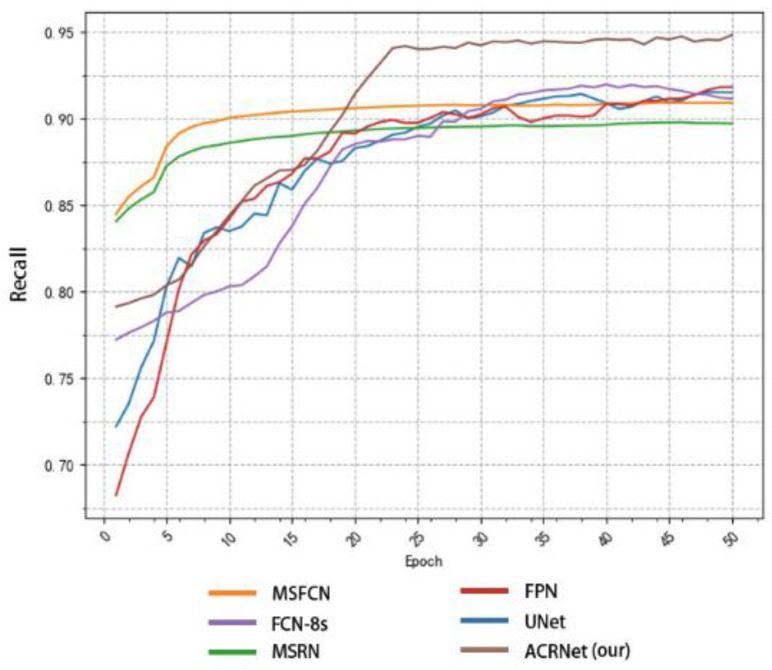
Variation of the recall of each model with the number of training epoch.

**Figure 10:**
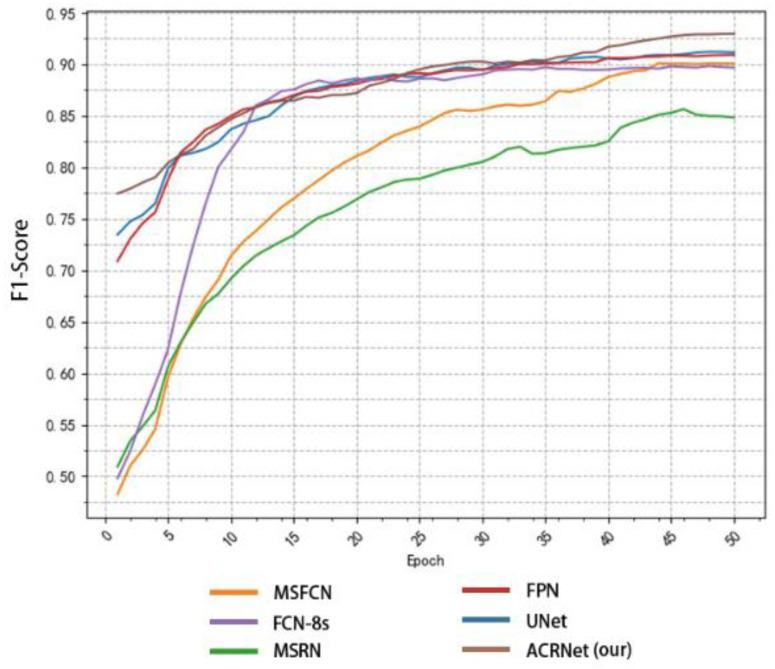
Variation of the F1-Score of each model with the number of training epoch.

In addition to comparing accuracy, we also made statistics on recall and F1-Score. Similarly, we took the first 50 epochs for research. Figure 9 shows the recall changes of different models with the change of epoch. MSRN and MSFCN improved rapidly in the early stage, but soon reached a relatively stable value and began to converge. Although ACRNet lagged behind several models at the beginning, there was a rapid improvement since about 20 epochs, and it has been maintained at a higher value than other models ever since. On the recall metrics, although ACRNet starts slightly slower than other models, it can get a more considerable improvement. Figure 10 shows the relation between F1-Score and the number of training epochs, which represents the robustness and stability of the model. ACRNet, FPN, U-Net, and FCN-8s have achieved good performance. ACRNet has less obvious advantages in the early stage of training, which is slightly higher than the other models. However, with the increase of the number of training epochs, according to the previous statistical results, we found that ACRNet can be 1% higher than the second-best model in later F1-Score comparison. From Figure 8, Figure 9 and Figure 10, we can observe that ACRNet model converges quickly and has good stability for each evaluation index such as accuracy, recall, and F1-Score. Therefore, it can be seen that the ACRNet combined with the composite decision-making approach makes the model have good stability.

**Figure 11:**
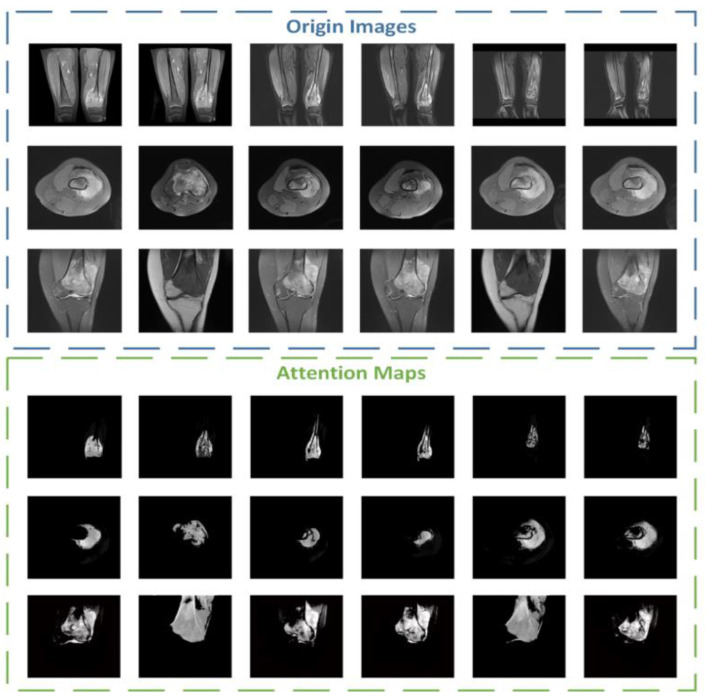
Some examples of attention maps from ACRNet.

During the experiment, we also conducted additional research on the attention part. We visualized the attention mechanism and superimposed the binary mask output by the middle layer with the input picture to obtain a more intuitive information range of attention focus, namely the attention map. The brighter the area, the more focused the attention. We selected 18 maps of different categories for comparison and display, and the results are shown in Figure 11.

## 5. Conclusions

In the article, the ACRNet was trained and tested by more than 4000 osteosarcoma MRI images. Through comparison and statistical analysis, we find that OMSAS has good prediction results in many indicators such as accuracy and recall. OMSAS not only improves the efficiency and accuracy of the model but also saves the cost with fewer parameters and shorter training time. The experimental results show that the segmentation model ACRNet in OMSAS has a better effect and more obvious advantages in osteosarcoma MRI image segmentation, and is suitable for application in developing countries with unbalanced medical resources distribution and poor medical care.

In the future, with further expansion and improvement of the dataset, we can continuously improve the prediction ability of the model, so as to detect the region of more complex osteosarcoma MRI images or even achieve similar good results on MRI of other cancers. At the same time, with the improvement of computing power and the need for later medical treatment, we can expand more modules in the model, such as outputting the size of the tumor area and predicting the future diffusion of tumor area, to provide more professional assistance for medical diagnosis.

## Figures and Tables

**Table 1:** Description of relevant symbols in the chapter.

Symbol	Represent Meaning
$p_{binary}$	The value of pixel *P*’s color in the picture after binarization
$\mu_{pixel}$	The average value of all pixels’ color values in the picture
$p_{norm}$	The regularized value of pixel P’s color value in the picture
$p_{\min},p_{\max}$	The highest color value and the lowest color value in a picture
$r_{j,k}$	The final output result of the (*j*, *k*)^th^ pixel on the picture
$p_{i,j,k}$	The value of the (*j*, *k*)^th^ pixel of *i*^th^ output picture
T(α,β)	Tversky Loss Function with *α* and *β* as variable
${\mathbb{R}}_{i},{\mathbb{Q}}_{i}$	The *i*^th^ pixel value of the prediction result and the true result, with a value of 0 or 1
ε	The value added to avoid the division of 0 by 0, with a value of 1 × 10^-8^

**Table 2:** Operating environment and some parameter settings.

Aspect		Concrete Content
Environment	Operating System	Ubuntu18.04.5 LTS
	CPU	Intel(R) Xeon(R) E5-2630L
	Memory	30G
	GPU	GTX 3060 12 GB
Parameter	Learning Rate	0.0001
	Epoch	460
	Experimenting Time	7 h~8 h

**Table 3:** Index comparison of different models in different image segmentation results.

Model	Accuracy	Recall	F1-Score	IOU	DSC	Params	SETT
UNet	0.9901	0.9294	0.9241	0.8672	0.8927	17.26 M	295
MSFCN	0.9917	0.9355	0.9059	0.8439	0.8929	23.38 M	306
MSRN	0.9880	0.9022	0.8662	0.8203	0.8832	14.27 M	288
FPN	0.9896	0.9238	0.9215	0.8518	0.8834	48.20 M	481
FCN	0.9891	0.9014	0.9213	0.8245	0.8763	134.3 M	783
Ours	0.9943	0.9408	0.9377	0.8848	0.9176	6.91 M	174

## Data Availability

Data used to support the findings of this study are currently under embargo while the research findings are commercialized. Requests for data, 12 months after publication of this article, will be considered by the corresponding author. All data analyzed during the current study are included in the submission.
